# Application of aptamers in regenerative medicine

**DOI:** 10.3389/fbioe.2022.976960

**Published:** 2022-08-29

**Authors:** Zhaohui Luo, Shimin Chen, Jing Zhou, Chong Wang, Kai Li, Jia Liu, Yujin Tang, Liqiang Wang

**Affiliations:** ^1^ Youjiang Medical University for Nationalities, Baise, Guangxi, China; ^2^ Guangxi Key Laboratory of basic and translational research of Bone and Joint Degenerative Diseases, Guangxi Biomedical Materials Engineering Research Center for Bone and Joint Degenerative Diseases, Department of Orthopedics, Affiliated Hospital of Youjiang Medical University for Nationalities, Baise, Guangxi, China; ^3^ Guangxi Botanical Garden of Medicinal Plants, Nanning, China; ^4^ Dalian Institute of Chemical Physics, Chinese Academy of Sciences, Dalian, China; ^5^ School of Mechanical Engineering, Dongguan University of Technology, Dongguan, Guangdong, China; ^6^ Academy of Orthopedics, Guangdong Provincial Key Laboratory of Bone and Joint Degeneration Diseases, The Third Affiliated Hospital of Southern Medical University, Guangzhou, China; ^7^ State Key Laboratory of Metal Matrix Composites, School of Material Science and Engineering, Shanghai Jiao Tong University, Shanghai, China

**Keywords:** aptamer, tissue regeneration, regenerative medicine, repair of tissue injury, transformation and application

## Abstract

Regenerative medicine is a discipline that studies how to use biological and engineering principles and operation methods to repair and regenerate damaged tissues and organs. Until now, regenerative medicine has focused mainly on the in-depth study of the pathological mechanism of diseases, the further development and application of new drugs, and tissue engineering technology strategies. The emergence of aptamers has supplemented the development methods and types of new drugs and enriched the application elements of tissue engineering technology, injecting new vitality into regenerative medicine. The role and application status of aptamers screened in recent years in various tissue regeneration and repair are reviewed, and the prospects and challenges of aptamer technology are discussed, providing a basis for the design and application of aptamers in long-term transformation.

## Introduction

Regenerative medicine is closely related to the treatment of injury and aging. The methods and strategies of regenerative medicine involve mainly stem cell technology, tissue engineering technology, and physical and chemical intervention (Fu, 2018; Edgar et al., 2020). Although regenerative medicine based on these strategies has achieved partial success in tissue and organ repair and regeneration, it is still subject to many defects. These defects include difficulty in obtaining exogenous stem cells, immunogenicity, tumorigenicity, low survival rate of exogenous stem cells (Tang, 2019), and high production cost of traditional protein active molecules. In addition, the environmental conditions for their biological activity are harsh (Perwein et al., 2018), and the application elements of tissue engineering technology are outdated (Atala and Forgacs, 2019; Matai et al., 2020).

Aptamers are a new class of ligand molecules that are manually screened by SELEX (systematic evolution of ligands by exponential enrichment) technology (Saito, 2021). They are short single-stranded DNA or RNA that can be folded into specific spatial structures. Moreover, they can bind to specific target molecules with high affinity and specificity through spatial complementarity, electrostatic attraction, van der Waals force, hydrophobic interaction, π-π stacking, and hydrogen bonding (Cai et al., 2018; Plach and Schubert, 2020). Compared with antibodies, aptamers have lower production cost, higher thermal stability, stronger renaturation after denaturation, easier chemical modification and labeling, wider target range, lower immunogenicity and cytotoxicity, and higher tissue penetration ability (Byun, 2021; Ni et al., 2021). In view of this, they have been widely applied in the biomedical field for testing, diagnosis, and targeted cancer therapy in the form of detection probes and a targeting medium (Zhang et al., 2021a; Mandal et al., 2021; Ni et al., 2021; Riccardi et al., 2021; Ma et al., 2022).

**FIGURE 1:**
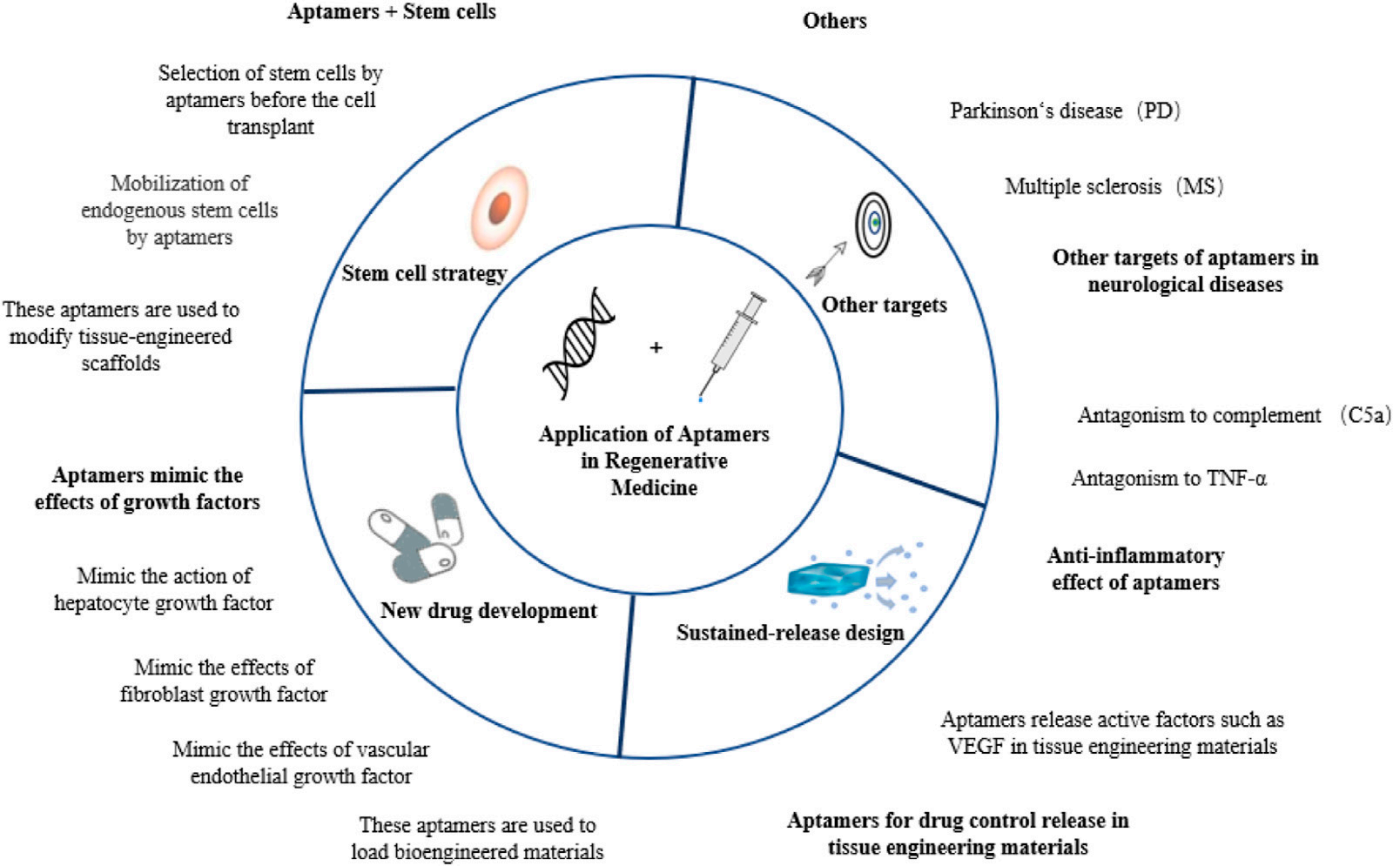
Schematic diagram of the partial application of aptamers in regenerative medicine.

Aptamers have recently been introduced into various regenerative medicine strategies, showing high application value in tissue repair and disease treatment. In this review, aptamers are classified according to their different modes of action, and their applications in tissue regeneration are reviewed. See Figure 1 for a general preview.

## Stem cell strategies combining aptamers in regenerative medicine

Stem cells can theoretically divide and proliferate infinitely. They exist widely in various tissues and can be derived and differentiated into various types of cells under specific environmental stimulation. Stem cells have become important resources in regenerative medicine because of their potency and growth capacity, especially for tissues with limited regenerative capacity, such as neural tissues (Gong et al., 2020) and muscle tissues (Roshanbinfar et al., 2021). Currently, stem cell strategies include mainly exogenous stem cell transplantation and endogenous stem cell mobilization and regulation. After arriving at the site of injury, stem cells from different sources play mainly a regenerative and repair role by integrating defects, secreting a variety of bioactive molecules, and partaking in immune regulation (Sarkar et al., 2021).

### Aptamer-based stem cell sorting technology can be used to obtain and purify target cells during stem cell transplantation

For exogenous stem cell transplantation, the sorting and collection of various stem cells is the primary problem. Classical cell sorting methods include immunomagnetic bead sorting (Laghmouchi et al., 2020), flow cytometry (Manohar et al., 2021), and solid surface sorting strategy based on cell adhesion differences (Durand, 2021). The first two methods are based mainly on the immunological principle of antibodies. However, during the sorting operations, cell viability and biological characteristics are often affected by antibody binding, fluid shear force, treatment of related enzymes, temperature changes, and electrical and chemical stimulation. Finally, they become unstable (Yao et al., 2020). For example, the combination of protein antibodies with stem cells can lead to changes in cell Stemness, changing the initial state of the cell and too intense ambient temperature, potential of hydrogen (pH) and electric field intensity can even induce cell degeneration and apoptosis, etc.

**FIGURE 2:**
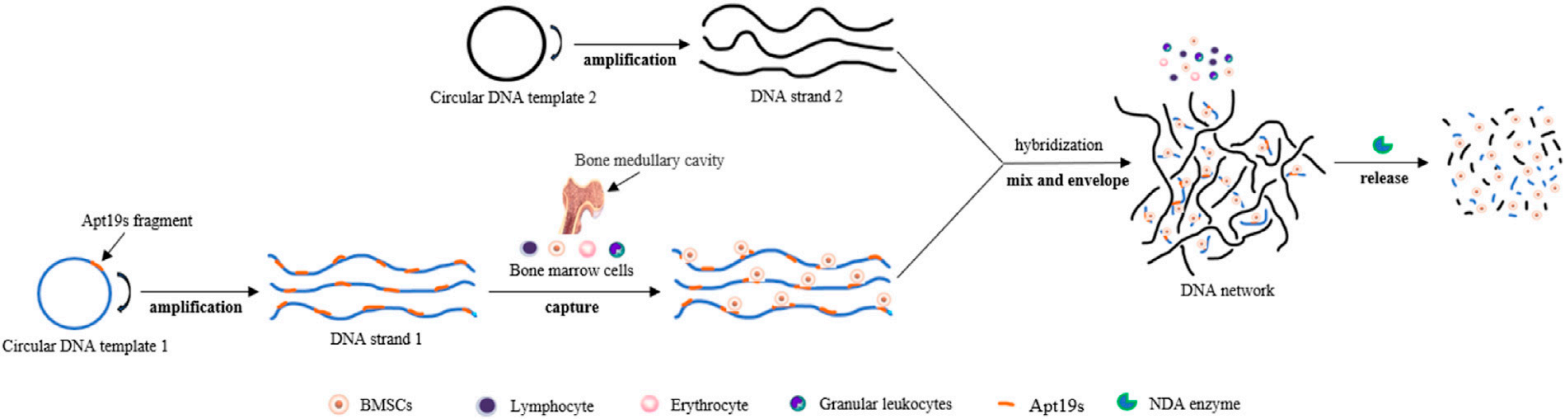
Schematic of a DNA cross-linking network designed for harvesting bone marrow mesenchymal stem cells. The process of capturing, enveloping, and releasing bone marrow stem cells (BMSCs). Capturing: DNA strand 1 was incubated with BMSCs, and cell capture was performed by aptamer Apt19s anchoring. Enveloping: Introducing DNA strand 2 into a cell solution containing DNA strand 1 triggers the formation of DNA networks. Releasing: The DNA network can be digested by DNA enzymes, which release BMSCs.

The emergence of SELEX technology based on cell or cell-specific markers has enabled aptamers to be used for sorting target cells. It has the advantages of low production cost, strong resistance to enzymatic hydrolysis, high purity, high efficiency, and little influence on cells. Over the past decade, a large number of aptamers with specificity and high affinity for various types of progenitor cells have been identified (Table 1). These include O-7 (a single-stranded DNA aptamer that binds human osteoblasts) (Guo et al., 2005; Guo et al., 2007), G-8 (an aptamer for binding adult mesenchymal stem cells (aMSCs) from pig bone marrow) (Guo et al., 2006; Schäfer et al., 2007), aptamer 36 (for CD31-positive cells in pig peripheral blood) (Hoffmann et al., 2008; Haller et al., 2015), specific binding aptamers of purified human CD31 extracellular domain (Strahm et al., 2010; Yoon et al., 2015; Kim et al., 2021a), specific RNA aptamers (L1-65, L2-2, and L3-3 binding to mouse embryonic stem cells (mESCs)) (Iwagawa et al., 2012), and DNA aptamers ((Aptamer-74) for osteoblast progenitors present in human jaw membrane cell population) (Ardjomandi et al., 2013). They have shown the ability to capture and enrich corresponding stem cells in bone marrow, whole blood, and other cell suspensions, suggesting their potential application in the collection of exogenous stem cells before transplantation and surface modification of biomaterials for tissue regeneration. Recently, de Melo et al. (2021) used adipose-derived stem cells (ASCs) from human adipose tissue and fibroblasts from skin tissue as screening targets and used Cell-SELEX and quantitative PCR technology to screen and identify an aptamer named Apta99. The aptamer can specifically recognize ASCs and shows a low affinity for fibroblasts, which is expected to be a powerful tool in ASC purification and therapeutic applications. Yao et al. (2020) also reported a cell capture technique beneficial to the survival of bone marrow mesenchymal stem cells (BMSCs). They introduced Apt19s (Hou et al., 2015), a single-stranded DNA aptamer with high specificity and affinity for BMSCs, into another cell-friendly DNA hydrogel system (Nam et al., 2021; Wu et al., 2021) to achieve a specific envelope and capture of stem cells during the formation of a hydrogel 3D DNA network. BMSCs showed uniform spatial distribution and good survival activity in this 3D network, and this system could achieve controlled release of captured cells under the digestion of nuclease. This principle is shown in Figure 2.

**TABLE 1:** Aptamers associated with stem cells and their experimental application.

Aptamer name	Aptamer type	Screening targets	Experimental application	References
O-7	Single-stranded DNA	SAOS-2 osteoblasts from human osteosarcoma	The surface of a cell culture plate with aptamer can directly and quickly capture osteoblasts from the cell suspension and enhance cell adhesion. The aptamer-modified titanium alloy surface can rapidly capture osteoblasts from the flowing suspension and enhance cell adhesion	(Guo et al., 2005; Guo et al., 2007)
G-8	Single-stranded DNA	aMSCs from porcine bone marrow	Isolation of aMSCs from porcine bone marrow; transplantation of ischemic myocardium	(Guo et al., 2006; Schäfer et al., 2007)
Aptamer 36	Single-stranded DNA	CD31-positive cells in peripheral blood of pigs	As a coating molecule, endothelial precursor cells (EPCs) with high expression of CD31 in pig blood can be captured *in vitro*. It can be used to isolate EPCs from pig bone marrow, and the isolated EPCs can be used for transplantation treatment in a pig myocardial infarction model	(Hoffmann et al., 2008; Haller et al., 2015)
AT-1	Single-stranded DNA	Purified extracellular domain of human CD31 molecule	It can be used to isolate EPCs from human umbilical cord blood, and the isolated EPCs can be used for transplantation therapy of hind limb ischemia in mice; a coating material for the surface of a vascular stent; vascularization of a bioengineered artificial liver	(Strahm et al., 2010; Yoon et al., 2015; Kim et al., 2021a)
L1-65、L2-2 and L3-3	RNA	Mouse embryonic stem cells (mESCs)	For the differentiation of mouse embryonic stem cells and other differentiated mouse cell lines; tracing the differentiation process of mESCs	Iwagawa et al. (2012)
Aptamer-74	Single-stranded DNA	Progenitor cells with osteogenic induction potential in human jaw membrane cells	Isolation of osteoblast progenitor cells from the human jaw bone periosteal cell population	Ardjomandi et al. (2013)
Apta99	Single-stranded DNA	Human adipose-derived stem cells (ASC)	It can be used to distinguish human fibroblasts from ASCs and purify ASCs	de Melo et al. (2021)
Apt19s	Single-stranded DNA	Human pluripotent stem cells	For the separation and purification of human embryonic stem cells; *in situ* tissue technique for homing mesenchymal stem cells promotes injury repair	(Hou et al., 2015; Hu et al., 2017; Wang et al., 2019a; Wang et al., 2019b; Kuang et al., 2019; Sun et al., 2021)
HM69	Single-stranded DNA	Human embryonic stem cells	*In situ* tissue technique for homing mesenchymal stem cells promotes injury repair	(Wang et al., 2019c; Yang et al., 2021a)

### Aptamers can capture and enrich endogenous stem cells at the site of tissue injury to promote tissue regeneration and repair

When tissue is damaged, the stimuli can activate the silent stem cells residing *in situ* to divide and proliferate for self-repair. Compared with exogenous stem cells, endogenous stem cells do not have problems with immunogenicity and origin (Yamanaka, 2020). Activating endogenous stem cells facilitate tissue repair. Traditional chemoattractant types have been used to activate and migrate endogenous stem cells and have gained recognition *in situ* tissue engineering techniques (Andreas et al., 2014).

In recent years, aptamers with *in vitro* cell capture ability have also been applied to *in situ* enrichment of endogenous stem cells and promote tissue regeneration. Apt19s (Hou et al., 2015; Wang et al., 2019a) is a single-stranded DNA aptamer derived from the SELEX sequence database of human pluripotent stem cells (PSCs). It shows high specificity and affinity binding to multiple gene-derived progenitor cells *in vitro*. Apt19s can be used as a powerful tool for the isolation, purification, and enrichment of PSCs. The binding target was identified as alkaline phosphatase-ALPL, a membrane protein type highly expressed on the surface of PSCs and its derived progenitor cells. The possibility of applying Apt19s to tissue engineering *in situ*, which facilitates the homing of stem cells, has been explored. In these designs (Hu et al., 2017; Wang et al., 2019b; Sun et al., 2021), the authors chemically coupled amino-modified Apt19s with activated carboxyl groups in various tissue-engineered scaffold components through the dehydration condensation reaction. They expected that the tissue-engineered scaffolds functionalized by this novel “cell catcher” could achieve a better repair effect after implantation at the injured site. The results showed that Apt19s on these scaffolds could recruit bone marrow mesenchymal stem cells and promote injury healing in a rat osteochondral knee joint defect model, rabbit osteochondral knee joint defect model, and critical size rat skull bone defect model. Kuang et al. (2019) produced a functional hydrogel by the covalent combination of Apt19s, which was modified by Acrydite (acrylic phosphoramidite) at the 5′ end of the nucleotide chain with an injectable composite hydrogel whose matrix was mainly composed of dimethylaminoethyl methacrylate (DMAEMA) and 2-hydroxyethyl methacrylate (HEMA). This hydrogel was also found to have obvious endogenous MSC recruitment when implanted into a rat femur bone defect model. Wang et al. (2019c) recently developed a new MSC-targeting aptamer HM69 using whole cell SELEX technology and constructed functional nanoparticles based on this aptamer. Such nanoparticles are essentially oligonucleotides formed by the end-to-end connection of nucleotide chains of the aptamer as independent units. Both HM69 and HM69 functional nanoparticles showed high specificity and affinity binding to MSCs *in vitro*, and such binding properties were evaluated to be superior to Apt19s. They applied the novel nanoparticles to treat a femur bone defect in a rat model and found that nanoparticles reached the bone defect via the circulation and then mediated *in situ* recognition, capture, and enrichment of MSCs. Yang et al. (2021a) also designed a 3D bio-printing dual-function bionic scaffold for *in-situ* cartilage regeneration based on the directional recruitment of MSCs by the HM69 aptamer. MSC cartilage differentiation was enhanced by transforming growth factor, which achieved good results in a rabbit full-layer cartilage injury model of a knee joint. In addition, a large number of designs have been reported that use aptamers to recruit and enrich endogenous repair factors at tissue injury sites (e.g.,vascular endothelial growth factor (VEGF) (Son et al., 2019), fractalkine (FKN) (Enam et al., 2017), etc.) and these designs also take full advantage of the binding specificity and adhesion between aptamers and target molecules.

In conclusion, aptamers play a role as a new cell sorting tool and a new cell chemoattractant and trapping agent in regenerative medicine stem cell strategies. Compared with the traditional application elements represented by active protein molecules, aptamers have more efficient cell sorting efficiency and better adaptability *in vivo*, and are easier to modify and connect with tissue engineering scaffolds. However, there are no reports on human experimental applications.

## Aptamer elements in a sustained-release hydrogel system

Aptamers with growth factor-like activity can be introduced into biomedical materials such as hydrogel to prolong the release time of aptamers, compared with using them alone. Moreover, aptamers can also be used as a controlled release device to control the release of other active molecules in sustained-release hydrogels.

### Active aptamers in hydrogels

**FIGURE 3:**
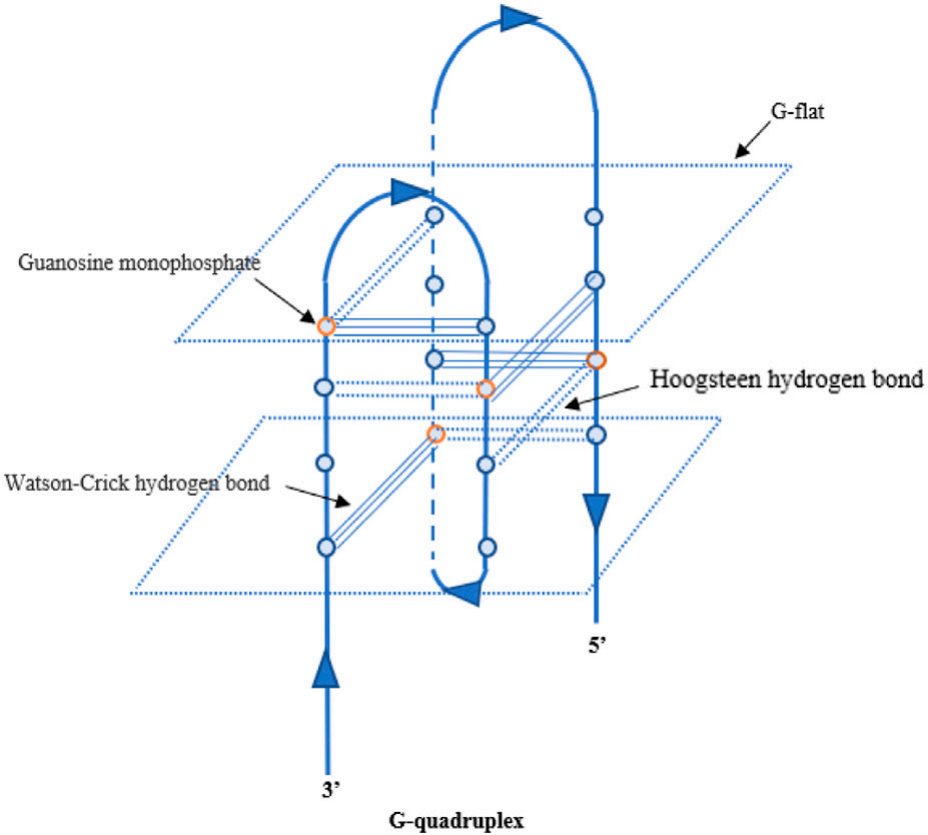
Schematic diagram of the G-quadruplex.

**FIGURE 4:**
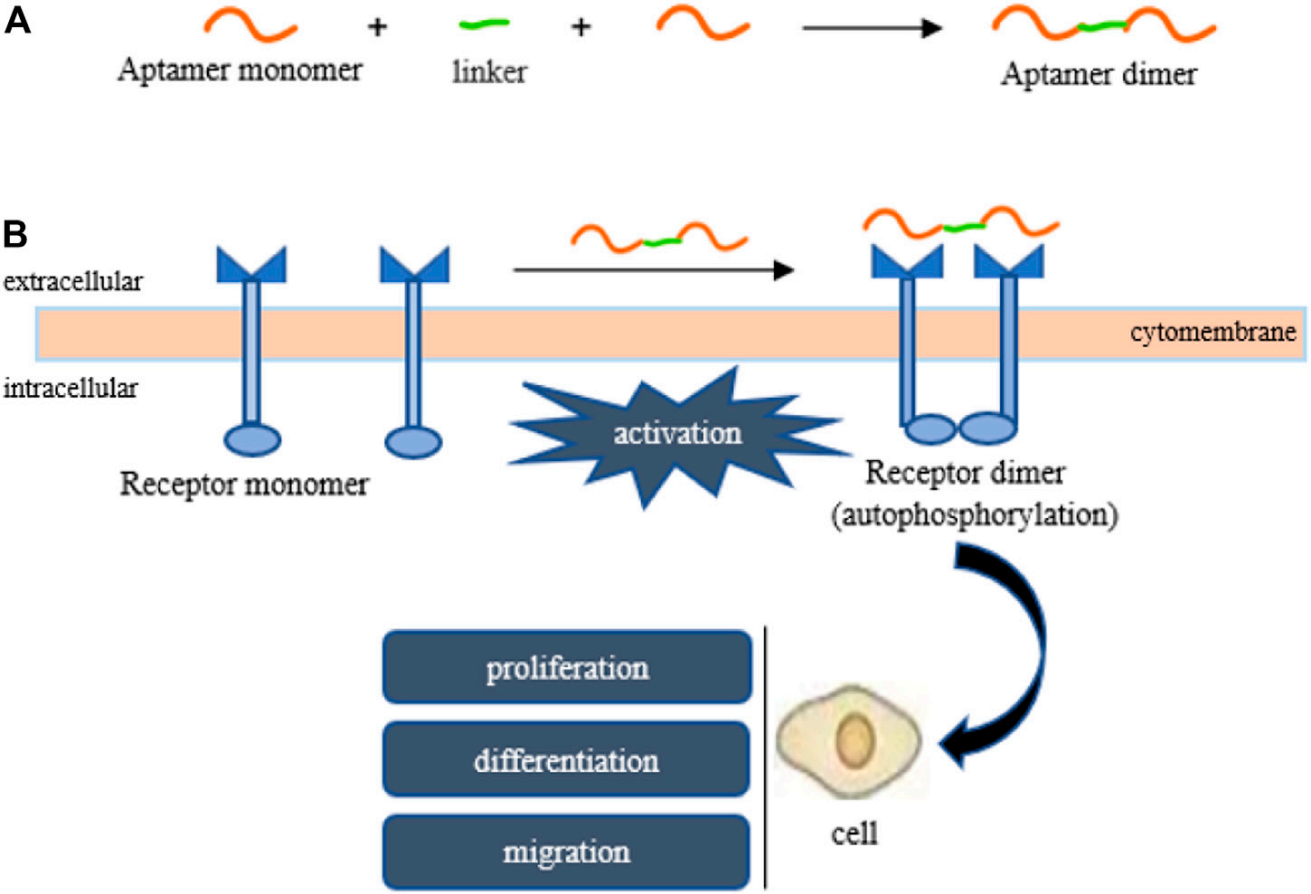
Aptamers mimic the activity of growth factors (HGF, bFGF, VEGF). **(A)** The formation of an aptamer dimer, **(B)** receptor-to-receptor dimerization and subsequent activation are both mediated by the aptamer dimer.

So far, some aptamers with growth factor-like activity have been screened to promote tissue regeneration. They have been combined with various hydrogel materials, showing a good application prospect in the field of tissue engineering. Ueki et al. (2016); Ueki et al. (2019); Ueki et al. (2020) obtained two aptamer dimers, ss-0 and TD0, that simulate the physiological effects of hepatocyte growth factor and basic fibroblast growth factor, respectively. The ss-0 is composed of two DNA aptamer monomers cross-linked by their complementary 5′-end sequences. These two monomers bind specifically and with high affinity to the receptor Met and have strong anti-nuclease stability. *In vitro* experiments have shown that ss-0 binds two Met receptor molecules to mediate intermolecular dimerization, including autophosphorylation of the receptor molecule, which stimulates cell migration and proliferation. After intravenous administration, the aptamer dimer showed ideal tissue distribution and pharmacokinetic characteristics, which are closely related to the G-quadruplex structure of the dimer sequence (see Figure 3). In a mouse model of outbreak hepatitis, ss-0 antagonizes the apoptosis of liver cells and alleviates the inflammatory response by specifically activating Met, proving its biosafety *in vivo*. TD0 specifically activates fibroblast growth factor receptor-1 (FGFR1). TD0 is directly linked by two SL38.2 DNA aptamers that target the extracellular domain of FGFR1 and can simulate the biological role of bFGF *in vitro*. This induces activation of FGFR1 dimerization and subsequent phosphorylation signal transduction pathways, supporting self-renewal and maintenance of stemness in induced pluripotent stem cells (iPSCs). It is noteworthy that SL38.2 also contains a guanine (G)-rich sequence that can form an antiparallel G-quadruplex structure in the physiological environment. This is considered to be key to its anti-nuclease stability and high-affinity binding to FGFR1. In 2015, Ramaswamy et al. (2015) reported a divalent aptamer (AptDivalent) assembled from two AptM80mer monomers, which specifically targets vascular endothelial growth factor receptor-2 (VEGFR2). Binding of AptDivalent to VEGFR2 on the surface of umbilical vein endothelial cells can activate the phosphorylation of receptors, activate the downstream Akt pathway, upregulate nitric oxide synthase content in endothelial cells, and ultimately promote endothelial cells to participate in the formation of new capillaries (all *in vitro*). James and Allen (2021) also reported the formation of a tubular phenotype *in vitro* in human umbilical vein endothelial cells (HUVECs) mediated by composite microfibers formed by the self-assembly of this aptamer dimer and collagen. Roy et al. (2021) introduced AptM80mer into a hyaluronic acid hydrogel and realized the functionalization of the hydrogel. They coupled the aptamer whose 5′ end of the nucleotide chain had been modified by an acrydite group to a thiolated-hyaluronic acid in the form of thioether bonds, which are formed under conditions of copolymerization. *In vitro* experiments based on HUVECs demonstrated that AptM80mer plays a similar role in endothelial cell activation and migration mediated by vascular endothelial growth factor (VEGF) in this hydrogel system and promoted angiogenesis. These studies indicate that the aptamer is expected to be an effective biochemical element for the vascularization design of engineering tissues (Figure 4).

### Aptamers can control the release of other active molecules in hydrogels

A novel sustained-release hydrogel system has been constructed by using aptamers as the affinity sites for proteins and bioactive oligonucleotides in hydrogels. This sustained-release hydrogel system has been proven beneficial to the long-term and flexible regulation of histopathological repair. In 2019, Abune et al. (2019) designed a double-aptamer-functionalized large-hole polyethylene glycol (PEG) hydrogel. They applied the same bonding method to introduce two DNA aptamers, modified by acrylate and targeting bFGF and VEGF, into the PEG network in the hydrogel system. This design also slowed the release rate of protein factors in the hydrogel and realized the long-term and stable release of active factors. The release mode of the two active factors mediated by this release platform synergistically promoted the migration of HUVECs and the regeneration of blood vessels in the chorioallantoic membrane (CAM) model *in vitro*. Zhao et al. (2019) also developed an aptamer-fibrin (Ap-Fn) hydrogel for controlling VEGF delivery, but the difference was the modification method of the aptamer. They first chemically coupled the mercaptosylated anti-VEGF aptamer with acrydite-modified natural fibrinogen in a copolymerization reaction between mercaptosylation and olefin to synthesize an aptamer-coupled fibrinogen macromolecule monomer. Compared with simple fibrinogen, the overall size and biological activity of this monomer hardly changed, and it could assemble and form a fibrin hydrogel under the action of thrombin and other clotting-related factors. It was proven that the anti-VEGF aptamer could significantly prolong the retention of VEGF in the Ap-Fn hydrogel, thus realizing the sustained release of VEGF. This improved VEGF release modality and promoted p-VEGFR2 expression and cell growth in HUVECs *in vitro*. Compared with the control hydrogels loaded with VEGF (hydrogels without aptamer modification or nonspecific aptamer modification), the Ap-Fn hydrogels loaded with VEGF could more effectively promote the generation of new blood vessels in the mouse skin trauma model and accelerate skin wound healing. The experiment by Juhl et al. (2019) also confirmed that the functional fibrinogen-aptamer did not lead to significant changes in gelation time, mechanical properties, or microstructure of the gel after gelation. However, the introduction of specific aptamers improved VEGF release kinetics in the aptamer-fibrin hydrogels (AFH), reducing the diffusion rate of VEGF compared with non-functional fibrin hydrogels (FH). The release time of VEGF was prolonged. In a mouse skull defect model of critical size, VEGF release mediated by the release platform functionalized by the aptamer increased the degree of vascularization *in vivo* and promoted osteogenesis more significantly than VEGF release mediated by the release platform without the introduction of the aptamer.

It can be seen from the above that aptamers could be used not only as a new bioactive ingredient to load hydrogels and other biological materials to promote tissue regeneration but also as a control device in biological materials to control the release process of other active ingredients. Screening more aptamer molecules for biological activity would be beneficial to regenerative medicine. In addition, we still need to pay more attention to the connection method between aptamers and biomaterial. We must also fully ensure that the biological activity of aptamers does not change after the connection, which is key to the future role of aptamers in the field of regenerative medicine.

## Antagonistic aptamers against pro-inflammatory mediators (plays a blocking role in inflammatory mediators)

The immune-inflammatory response plays an important role in the repair of organ and tissue injury. A large number of therapeutic strategies targeting inflammatory factors or inflammatory factor receptors have been attempted for tissue regeneration. As a new type of ligand molecule, aptamers can influence the biological activities mediated by the target after binding with their targets. As a result, a number of aptamers directly involved in the regulation of inflammation have been developed. These aptamers can specifically bind inflammatory mediators, including interleukin-2 (IL-2), IL-6, IL-10, IL-11, IL-17, IL-32, transforming growth factor-β (TGF-β), tumor necrosis factor-α (TNF-α), interferon-γ (IFN-γ), chemokines CCL2, IP-10, and some related receptors (Boshtam et al., 2017), and even some inflammatory autoantigens (Mor-Vaknin et al., 2017; Cao et al., 2021), etc. Here, we focus on several aptamers with immunomodulatory activity developed in recent years. The difference is that they have been studied *in vivo*, and the types of aptamers that regulate complement activity have been developed.

### Aptamers that bind TNF-α or TNF receptor

TNF-α is an important member of the TNF superfamily and is derived mainly from monocytes and macrophages. Other cells such as T and B lymphocytes, natural killer cells, mast cells and endothelial cells, neutrophils, smooth muscle cells, and cardiomyocytes can also release this cytokine. TNF-α is also one of the most potent cytokines found in mammals. Its pleiotropy is reflected mainly in its ability to bind both TNFR1 and TNFR2. The binding of TNF-α to TNFR1, one receptor type, leads to a negative inflammatory process or apoptosis. The binding of TNF-α to TNFR2 produces positive repair effects, such as maintaining cell survival and promoting tissue regeneration. These two opposite biological effects of TNF-α are closely related to the mitogen-activated protein kinase (MAPK) signaling pathway (Tseng et al., 2018; Zhang et al., 2020).

The inhibitors represented by TNF-α monoclonal antibodies are thought to effectively block the destructive effect of TNF-α (Mitoma et al., 2018). According to the same principle, a new class of aptamer-based TNF-α inhibitors has been developed. In 2013, Orava et al. (2013) identified a DNA aptamer VR11 that specifically recognizes human recombinant TNF-α. *In vitro* experiments showed that the aptamer could block the binding process between TNFα and TNFα receptor, thus inhibiting the cytotoxicity of TNF-α, which was similar to the action of TNF-α antibody. Kim et al. (2021b) introduced VR11 into a metal nanoparticle that is often used as a carrier material in biomedical applications. This not only enhanced the stability of aptamers but also played a role in capturing TNF-α at the inflammatory site *in vivo* and inhibiting the inflammatory response. Lai et al. (2019) developed a novel TNF-α targeting aptamer (aptTNF-α) and its PEG derivative (aptTNF-α-PEG), which showed good affinity for human/mouse TNF-α *in vitro*. In addition, targeted inhibition of TNF-α was also shown in acute lung injury and acute liver failure mouse models with high TNF-α expression. *In vivo* application of aptTNF-α/aptTNF-α-PEG reduced the degree of the acute inflammatory response in both models and promoted early regeneration of liver tissue. These studies indicate the potential of aptamers as non-immunogenic oligonucleotide inhibitors against TNFα.

Although TNF-α inhibitors inhibit the negative effects of TNF-α, they also eliminate the positive contribution of TNF-α to inflammation. To solve this problem, Zhang et al. (2020) screened specific aptamers for the inflammatory receptor TNFR1 downstream of the TNF-α signaling pathway, hoping to inhibit inflammation and improve the therapeutic effect by selectively inhibiting TNFR1. So far, they have obtained eight aptamers with high affinity for TNFR1 and two with low affinity for TNFR2. However, there are no relevant experimental data in cells and zoology.

### Aptamers antagonizing complement (aptamers binding to complement)

Among these aptamers, NOX-D20 (Hoehlig et al., 2013), which binds and blocks complement C5a, has been studied the most. C5a is considered a potent anaphylactic toxin, and excessive C5a demonstrates a cascade of inflammatory destruction (Wood et al., 2018). NOX-D20 is a mirror aptamer synthesized from an unnatural L-oligonucleotide known as Spiegelmers. *In vitro* experiments have shown that NOX-D20 could block the release of elastase from polymorphonuclear leukocytes induced by C5a and the chemotaxis of CD88 expression cell lines. These two pathological events are closely related to pro-inflammation. In rodent sepsis models induced by cecal ligation and puncture, it showed therapeutic effects of reducing inflammation and organ damage, preventing the destruction of the vascular endothelial barrier, and improving the survival rate. The specific binding and inhibitory activity of NOX-D20 on C5a has been proven to be closely related to the G-quadruplex structure of its molecular structure (Yatime et al., 2015). In recent years, people have developed ways of using and treating NOX-D20. Li et al. (2019) combined a framework of nucleic acid (FNA) (Zhang et al., 2021b; Zhang et al., 2021c; Gao et al., 2022) in the form of a DNA origami nanostructure to improve the stability and targeting of NOX-D20 *in vivo*. They used bipyramidal FNA as a drug delivery platform to deliver NOX-D20 to treat cerebral ischemia-reperfusion injury and achieved good results. Zhang et al. (2021d) conjugated NOX-D20 with cerium dioxide nanoparticles (Ni et al., 2019) with active oxygen scavenging activity and formed the complex Ceria@Apt, which not only integrates the anti-inflammatory advantages of both but also makes full use of the liver absorption effect of nanomaterials. This composite material shows great application potential in the repair of liver ischemia-reperfusion injury.

In general, although various inhibitors against C5a have been extensively studied, the types of inhibitors against downstream c5A-related signaling molecules have not been reported.

## Aptamer types that act on other pathological targets in neurological diseases

In recent years, studies on the types of aptamers involved in treating several neurological diseases (represented by multiple sclerosis and Parkinson’s disease) have become increasingly mature. These have provided ideas for developing new aptamer-based drugs, which are also introduced here.

### Aptamers associated with multiple sclerosis

Inspired by the fact that a natural IgM antibody can promote myelin regeneration after binding to oligodendrocytes both in a mouse multiple sclerosis (MS) model infected with Theiler’s myeloencephilitis virus (TMEV) (Dubik et al., 2021) and in a mouse model of focal demyelination induced by lysophosphatidylcholine (Bieber et al., 2002), Nastasijevic et al. (2012) identified a small molecule single-stranded DNA aptamer 3064, or LJM-3064 (Wilbanks et al., 2019). This aptamer is smaller than the IgM monoclonal antibody by *in vitro* screening and targets the crude extract of myelin from SJL mice (the myelin preparation is a crude mixture of proteins and lipids). The aptamer contains a guanylate-rich domain and has shown high affinity and specific binding ability to myelin basic protein (MBP) subtypes *in vitro*. Previous studies (Bose, 2021) have shown that the sequence encoded by MBP exon 2 in the MBP subtype is an effective target for myelin regeneration. Their further study found that a streptavidin tetramer could mediate the coupling of four aptamers. This formed the tetramer aptamer complex Myaptavin-3064, which mimics the activity of polyvalent antibodies (Fereidan-Esfahani et al., 2020). The biological stability of the aptamer complex was enhanced compared with that of the aptamer monomer. Intraperitoneal injection of demyelinated mice infected with TMEV enhanced myelin regeneration in the mouse model, while aptamer monomers did not.

In later studies, Smestad and Maher (2013) verified that 26 nucleotides at the 5′ terminal of LJM-3064 (guanylate-rich region) were involved in forming the G-quadruplex through intramolecular folding. They also proved the univalent ion-dependent conformational switching of the G-tetrahedron structure in the physiological environment. Namely, in the absence of sodium and potassium ions, LJM-3064 adopts an antiparallel G-tetrahedron structure. When transferred into a buffer that mimics low potassium concentrations in tissue fluid and plasma, LJM-3064 rapidly transitions into a thermodynamically more stable parallel chain G-tetrahedron conformation. Their data also showed that the parallel G-quadruplex structure of the LJM-3064 monomer still existed in the tetravalent complex Myaptavin-3064. Perschbacher et al. (2015) used quantitative PCR to analyze the effect of the nucleotide sequence structure of LJM-3064 on its pharmacokinetics and the pharmacokinetic characteristics of various LJM-3064 derivatives produced by different modification methods. The results suggest that LJM-3064s tissue penetration and anti-enzymatic activity may be related to the G-rich sequence in its composition structure, and the polyvalent property of MyAPtavin-3064 may more affect the biological activity of myelin regeneration. Wilbanks et al. (2019) truncated and optimized a DNA aptamer LJM-5708 containing only 20 nucleotides based on LJM-3064, which retained the rich G sequence of the G-quadruplex structure formed by the original aptamer. The polyvalent aptamer complex formed by LJM-5708 showed enhanced myelin-binding properties compared with the LJM-5708 monomer, and also showed significant binding ability to human oligodendrocytes cultured *in vitro*. The study also pointed out that the absence of the G-quadruplex formation of the LJM-5708 aptamer results in the loss of myelin binding function of the polyvalent aptamer complex. Heider et al. (2018) designed a streptavidin-free aptamer polymer (named 3064-4WJ-LNA) on the basis of previous work, which is expected to improve immunogenicity.

In addition to studying the role of the LJM-3064 aptamer alone in MS myelin regeneration therapy, LJM-3064 was also conjugated to a mesenchymal stem cell-derived exosome with immunomodulatory functions. Two synergistic biological effects of myelin regeneration and anti-inflammation were achieved in the multiple sclerosis mouse model induced by a myelin oligodendrocyte glycoprotein, which expanded the application of the LJM-3064 aptamer (Hosseini et al., 2019).

In 2020, Fereidan-Esfahani et al. (2020) used flow cytometry and immunocytochemistry to determine the cell-binding properties of Myaptavin-3064 *in vitro*. Myaptavin-3064 shows relatively specific binding to the human oligodendroglioma (HOG) cell line. In particular, the differentiated HOG shows enhanced binding ability with no affinity for lung (L2) or kidney (BHK) cell lines. In addition to HOG cells, Myaptavin-3064 could bind to adult rat oligodendrocytes (OLs) but not to primary cortical cells derived from embryonic mice. The cell-binding properties of Myaptavin-3064 indicate that the target of Myaptavin-3064 exists in the more mature myelin sheath and also support the hypothesis that Myaptavin-3064 induces myelin regeneration through OL binding.

The migration of autoreactive immune cells across the BBB (blood-brain barrier) is considered to be a pathological feature of MS, and it plays a key role in the formation of MS demyelinating plaques (Goverman, 2009). The α4-integrin family mediates the adhesion, exudation, and migration of these inflammatory cells (Mitroulis et al., 2015). Based on the importance of α4-integrin in the pathogenesis of MS, a blocking antibody against α4-integrin, natalizumab, has been used for phase 3 clinical observation in patients with secondary progressive multiple sclerosis (Kapoor et al., 2018). According to a similar concept of action, Kouhpayeh et al. (2019) screened a specific single-stranded DNA aptamer with a high affinity for α4-integrin, which is expected to play a therapeutic effect similar to natalizumab on MS.

### Aptamers associated with Parkinson’s disease

The α-synuclein (α-syn) is a small natural non-folding protein containing 140 amino acids, existing mainly in the presynaptic terminal in neurons (Burré et al., 2018). In a physiological state, α-syn is a kind of disordered protein in nature. Once stimulated by various injury factors, it tends to form oligomers and aggregates, which may produce toxic effects on neurons through mitochondrial injury, axon transport dysfunction, inflammation, and other mechanisms (Shahnawaz et al., 2020). More importantly, once the abnormal α-syn protein aggregates are absorbed by other normal cells, they can secondarily cause the same pathological process as other normal α-syn proteins in the recipient cells. This leads to intercellular infection and diffusion (Valera and Masliah, 2016), which are important in the formation and progression of PD. Zheng et al. (2018) attempted an aptamer strategy to regulate α-syn toxicity by screening two aptamers with high specificity and affinity for α-syn protein (named F5R1 and F5R2, respectively) using optimized SELEX. Both aptamers could effectively reduce α-syn accumulation in extracellular and intracellular domains, especially in cells. When delivered into human neuroblastoma cells and primary neurons by a carrier peptide, the targeted binding of the two aptamers to α-syn inhibits the formation of pathogenic aggregates and mediates intracellular degradation of α-syn, saving mitochondrial dysfunction and cell damage caused by α-syn overexpression. Ren et al. (2019) studied the *in vivo* effect of these aptamers on PD-related neuropathological defects. They used a neuron-targeting exosome (Alvarez-Erviti et al., 2011) as a loading vehicle for these therapeutic aptamers, achieving efficient delivery of aptamers to neurons *in vivo* and *in vitro*. When co-cultured with primary neurons, aptamer-loaded exosomes significantly reduced the formation of intracellular secondary α-syn insoluble aggregates induced by α-syn fibrils and saved synaptic protein loss and neuronal death. Intraperitoneal injection of this compound in a PD mouse model showed a significant reduction in the pathological aggregates of α-syn in the brain and a significant improvement in the associated motor dysfunction, further confirming the therapeutic ability of these aptamers *in vivo*.

### Aptamers associated with Nogo-66 receptor

Myelin-derived inhibitors, represented by Nogo, myelin-associated glycoprotein (MAG), and oligodendrocyte myelin glycoprotein (OMgp), inhibit the growth of neuronal processes by binding to the Nogo-66 receptor (NgR). Using the NgR as the screening target, Wang et al. (2010) obtained a set of RNA aptamers. *In vitro* experiments showed that the binding properties of these aptamers to NgR did not affect neurite growth but could competitively block the inhibition of neurite growth by these three inhibitors. Agrawal et al. (2020) designed a sustained-release hyaluronic acid hydrogel modified by several antisense oligonucleotides, and realized the slow and sustained release of these highly therapeutic aptamers by using the complementary pairing relationship between antisense oligonucleotide sequence and NgR targeted aptamers to varying degrees. It had the same protective effect on the neuronal processes damaged *in vitro*.

## Summary and prospects

Regenerative medicine makes full use of various biological techniques and engineering principles to provide better and faster repair of diseases. In the past, stem-cell strategies, tissue engineering techniques, and physicochemical techniques have been applied to regenerative medicine, which has achieved remarkable results. However, in the actual process of operation, people are still faced with many problems, such as difficulties in obtaining stem cells and limited means of regulating cell biological behavior. In addition, there is a lack of development mode and type of new chemical preparations with biological activity, as well as a scarcity of tissue engineering technology application factors.

Aptamers are a kind of ligand nucleic acid molecule with similar binding properties to antibodies. The binding targets are varied, including inorganic ions, amino acids, antibiotics, peptides, proteins, sugars, nucleotides, and their derivatives (Plach and Schubert, 2020). At the same time, these targets can be used as specific markers of bacteria (Yang et al., 2021b), viruses (Lu et al., 2021), cells (Lin et al., 2021), and other living organisms. Aptamers are widely used in various fields of biomedicine, especially in testing, diagnosis, and targeted therapy of cancer, due to their different binding effects with different targets. In a word, aptamers can be called “artificial high quality nucleic acids” because of their universality of application purposes, wide range of action targets, flexibility of action time and space, and diversity of action forms.

From the perspective of tissue regeneration, this article on aptamers in related research in recent years has continued the review and summary. The results show that aptamers can be used alone or crossed with each other through various technical means, such as stem cell technology, biological engineering materials such as hydrogels, nanomedicine, immunotherapy, and so on. Therefore, they show strong plasticity and a broad transformation prospect for applied research in regenerative medicine. At the same time, this paper also provides a reference for the long-term design and utilization of aptamers. Due to the limited amount of literature and the inability to categorize and report, we could not summarize the modification methods of aptamers for therapeutic application.

Many problems still need to be solved for the practical application of aptamers. These include further cost control, development of new aptamer types, improvement in screening efficiency, creation of modifications that improve pharmacokinetics and enhance biological activity *in vivo*, optimization of connection methods with other biomedical materials, and advancing zoological and human experiments. We believe that aptamers can better serve us after these problems have been solved.

## Data Availability

The original contributions presented in the study are included in the article/Supplementary Material, further inquiries can be directed to the corresponding authors.
